# Satellite telemetry tracks flyways of Asian Openbill storks in relation to H5N1 avian influenza spread and ecological change

**DOI:** 10.1186/s12917-018-1683-x

**Published:** 2018-11-16

**Authors:** Parntep Ratanakorn, Sarin Suwanpakdee, Witthawat Wiriyarat, Krairat Eiamampai, Kridsada Chaichoune, Anuwat Wiratsudakul, Ladawan Sariya, Pilaipan Puthavathana

**Affiliations:** 1 Department of Clinical Science and Public Health, Faculty of Veterinary Science, Mahidol University, Nakhon Pathom, 73170 Thailand; 2 The Monitoring and Surveillance Center for Zoonotic Diseases in Wildlife and Exotic Animals, Faculty of Veterinary Science, Mahidol University, Nakhon Pathom, 73170 Thailand; 3 Department of Preclinic and Applied Animal Science, Faculty of Veterinary Science, Mahidol University, Nakhon Pathom, 73170 Thailand; 4 Department of National Parks, Wildlife and Plant Conservation, Ministry of Natural Resources and Environment, Bangkok, 10900 Thailand; 5 Center for Research and Innovation, Faculty of Medical Technology, Mahidol University, Nakhon Pathom, 73170 Thailand; 6 Department of Microbiology, Faculty of Medicine Siriraj Hospital, Mahidol University, Bangkok, 10700 Thailand

**Keywords:** *Anastomus oscitans*, Asian Openbill, H5N1 highly pathogenic avian influenza (H5N1 HPAI), Satellite telemetry, Flyway

## Abstract

**Background:**

Asian Openbills, *Anastomus oscitans*, have long been known to migrate from South to Southeast Asia for breeding and nesting. In Thailand, the first outbreak of H5N1 highly pathogenic avian influenza (HPAI) infection in the Openbills coincided with the outbreak in the poultry. Therefore, the flyways of Asian Openbills was determined to study their role in the spread of H5N1 HPAI virus to poultry and wild birds, and also within their flocks.

**Results:**

Flyways of 5 Openbills from 3 colonies were monitored using Argos satellite transmitters with positioning by Google Earth Programme between 2007 and 2013. None of the Openbills tagged with satellite telemeters moved outside of Thailand. Their home ranges or movement areas varied from 1.6 to 23,608 km^2^ per month (95% utility distribution). There was no positive result of the viral infection from oral and cloacal swabs of the Openbills and wild birds living in the vicinity by viral isolation and genome detection during 2007 to 2010 whereas the specific antibody was not detected on both Openbills and wild birds by using microneutralization assay after 2008. The movement of these Openbills did not correlate with H5N1 HPAI outbreaks in domestic poultry but correlated with rice crop rotation and populations of the apple snails which are their preferred food. Viral spread within the flocks of Openbills was not detected.

**Conclusions:**

This study showed that Openbills played no role in the spread of H5N1 HPAI virus, which was probably due to the very low prevalence of the virus during the monitoring period. This study revealed the ecological factors that control the life cycle of Asian Openbills.

**Electronic supplementary material:**

The online version of this article (10.1186/s12917-018-1683-x) contains supplementary material, which is available to authorized users.

## Background

Asian Openbills or Asian Open-billed storks, *Anastomus oscitans*, are large wading birds in the family *Ciconiidae* [1]. The birds get their name from the natural open space between the curved inner surfaces of the mandibles in adults [2]. This gap between the mandibles increases with age. Openbills are migratory birds which generally move between South Asian countries (India, Sri Lanka, Bangladesh) and Southeast Asian countries (Myanmar and Thailand) [3, 4]. Using ring bands, McClure and Kwanyen in 1973 suggested that Asian Openbills migrate from Bangladesh to Thailand for breeding and nesting [1, 3]. In Thailand, Asian Openbills gather together during the breeding season, around November to February, which are cold months of the year. These birds are colonial breeders with several nests being built in the same tree [5]. Openbills forage in marshes and paddies and feed on mollusks, frogs, crabs, aquatic animals, snakes and giant insects [5, 6]. In Thailand, their favorite food is apple snails [5, 7], including *Pomacea canaliculata* and *P. insularum* [7]. The gap between the mandibles allows the birds to hold and carry snails easily [8].

The outbreaks of H5N1 highly pathogenic avian influenza (HPAI), occurred in Thailand in January 2004 and resulted in enormous economic losses of poultry and several human deaths. With restricted control strategy, no human case was found after July 2006 [9], while no outbreak occurred in poultry farm after 2008 [10]. At the early event of the outbreak, a lot of Asian Openbills were found dead in the rice paddy fields together with free-ranging ducks. In 2004, the prevalence of H5N1 HPAI virus infection in the Openbills of unknown health status was as high as 26.67% (95% confidential interval-CI: 13.7–39.6) as determined by virus isolation and viral genome detection. The prevalence decreased to 0.91% (95% CI: 0.1–1.7) in 2005, and 0% in 2006 and 2007 [11].

Initially, the Openbills was suspected of bringing in the virus along with their migration from Bangladesh to Thailand. Therefore, depopulation of Openbills was proposed to be an approach for avian influenza (AI) control. However, this opinion was stopped based on the information that there was no occurrence of H5N1 HPAI outbreak in South Asia at that time. According to the Office International des Epizooties (OIE) [10], the first outbreak of this virus in South Asia occurred in Bangladesh in March 2007. The new opinion speculated that the Openbills might get the H5N1 virus infection from grazing ducks and/or resident birds that shared the foraging habitats in rice paddy fields.

Our field observation revealed that the flock of Openbills returned to the old nesting places in early November every year, and by the end of February, the flocks disappeared from the nesting areas or markedly decreased in population size. No further information exists on where the birds go after February, and the role of Openbills in H5N1 AI spread has not been elucidated. This study used satellite telemetry in combination with field surveys and virological assays to monitor the flyways of Openbills and investigate the possible spread of H5N1 HPAI virus within the flocks and to other animal species living in the vicinity in Thailand.

## Results

### Biographic data and monitoring periods of Openbills

Biographic data of the 5 Openbills tagged with satellite transmitters are shown in Table 1. Their flyways were monitored for the duration of 1–5 years. During the cold months of the year (November–February), the Openbills gathered together for nesting, breeding and egg-laying, and the colonies became overcrowded with birds. About 50 days after hatching, the baby birds became juveniles, and the flocks left their nests to other foraging areas in February to March. The Openbills returned to their old nesting areas in the next breeding season. None of the birds flew from Thailand to other countries during the monitoring period (Table 1).

**Table 1:** Biographic and tracking period of the Asian Openbills monitored by satellite telemetry

Bird No.	Transmitter No.	Sex	Body weight (kg)	System of satellite transmitter	Study site	Transmitter tagging date	Time at signal loss from monitoring	Tracking period
1	74793	Male	1.40	35 g, Solar PTT-100	Nakhon Pathom	02/25/2007	05/30/2008	1 year 3 months
2	74794	Female	1.50	35 g, Solar PTT-100	Nakhon Sawan	01/03/2007	09/06/2012	5 years 6 months
3	74799	Female	1.50	45 g, GPS PTT-100	Nakhon Sawan	01/03/2007	01/25/2010	3 years 1 month
4	74800	Male	1.40	45 g, GPS PTT-100	Nakhon Pathom	02/25/2007	12/18/2008	1 year 10 months
5	30123	ND	ND	35 g, Solar PTT-100	Phitsanulok	07/21/2009	09/16/2013 (Stopped tracking)	More than 4 years 2 months

### Movement areas of the Openbills

The data used in this study are available on Movebank (movebank.org, study name “Asian Openbill tagging with satellite telemetry in Thailand, 2007-2013”) and are published.

in the Movebank Data Repository [12].

**Fig. 1:**
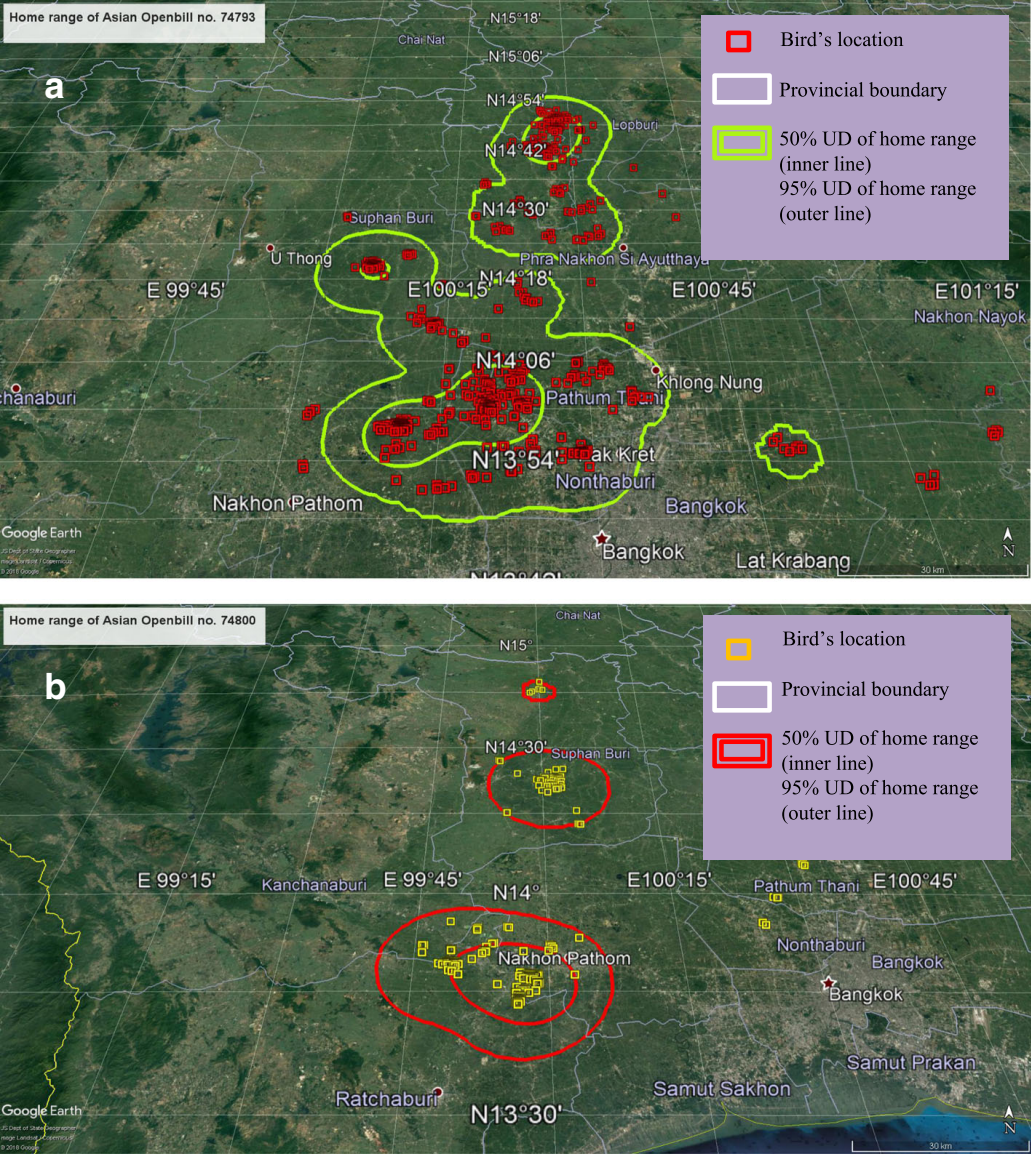
The home ranges of Asian Openbills using satellite telemetry tracking (**a**) animal ID 73793 (**b**) animal ID 74800

The Openbill ID numbers 74,793 and 74,800 nested and formed colonies at the study site in Nakhon Pathom province. Both birds were tracked foraging in several provinces in Central Thailand. The average home-range area of bird 74,793 was 1564 km^2^/month (range 6.6–11,642: 95% Utility Distribution - UD); and it was 2477 km^2^/month (range 2.5 to 23,608: 95% UD) for bird 74,800 (Tables 1 and 2). Unfortunately, the signals from satellite transmitters on these two Openbills lost after they flew back to their old nested area in the second season. Their habitat home ranges were relatively wide around Chao Phraya River basin where a main river of Thailand is. That area presented mainly as the rice farming and the agricultural area (Fig. 1a and b).

**Table 2:** The movement areas of Asian Openbills as tracked by satellite telemetry

Bird ID	Home range area/month (km^2^), 95%UD			Movement areas by provinces
	Maximum	Minimum	Average	
74793	11,642	6.6	1564	Nakhon Pathom, Ratchaburi, Nonthaburi, Phra Nakhon Si Ayutthaya, Suphan Buri, Ang Thong, Saraburi, Sing Buri, Lop Buri, Chai Nat, Nakhon Sawan and Phichit
74800	23,608	2.5	2477	Nakhon Pathom, Ratchaburi, Suphan Buri, Nakhon Sawan and Phichit
74794	752	3.4	76	Nakhon Sawan, Phichit, Phetchabun and Phitsanulok
74799	4481	1.6	414	Nakhon Sawan, Phichit, Chainat, Sing Buri and Ang Thong
30123	2833	4.6	360	Phitsanulok, Phichit, Nakhon Sawan, Phetchabun and Nakhon Pathom

**Fig. 2:**
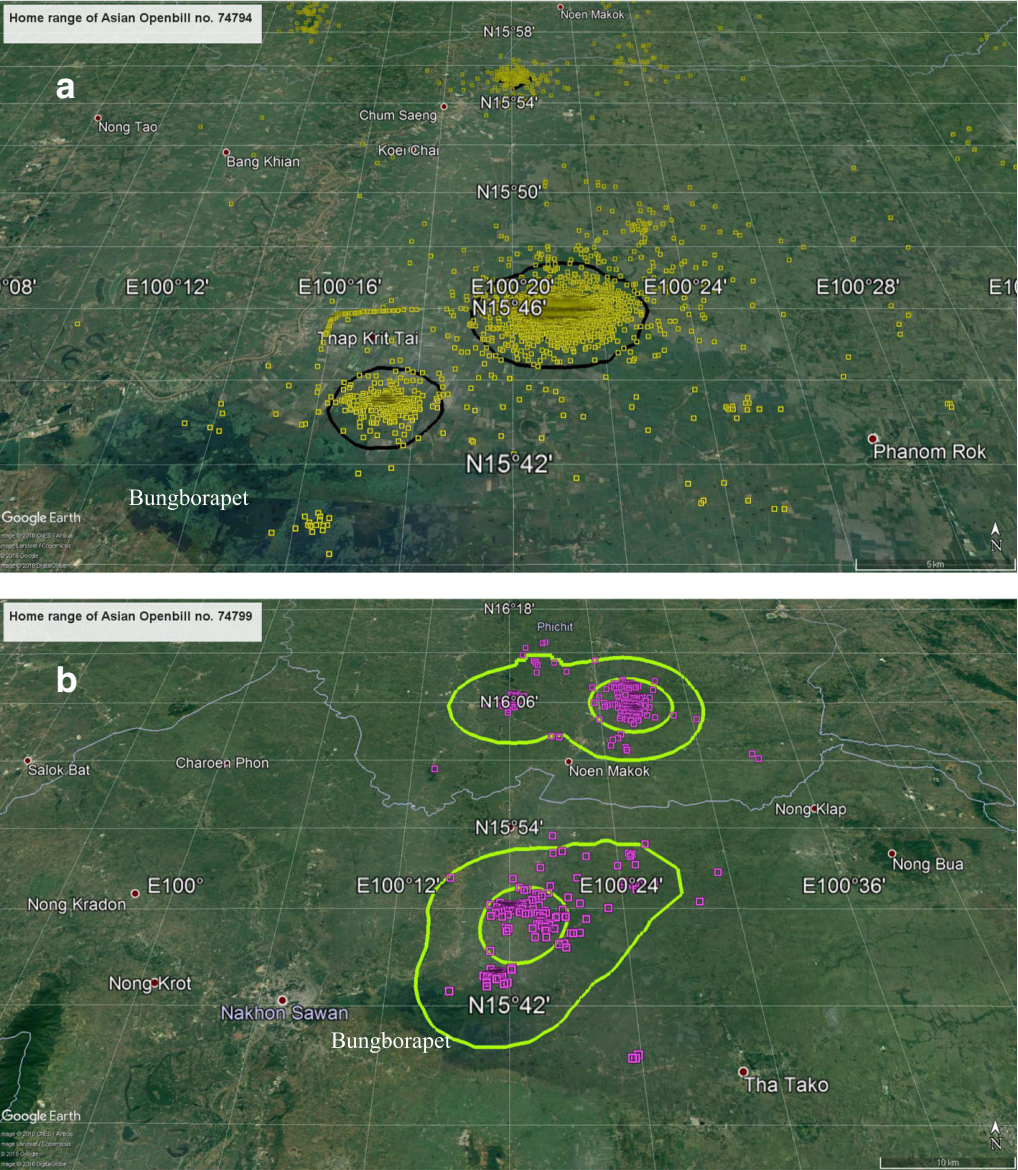
The home ranges of Asian Openbills using satellite telemetry tracking (**a**) animal ID 73794 (**b**) animal ID 74799

The Openbill ID numbers 74,794 and 74,799 from the Nakhon Sawan study site did not forage far from their origin and had average home-range areas of 76 km^2^/month (range 3.4 to 752: 95% UD) and 414 km km^2^/month (range 1.6 to 4481: 95% UD), respectively. The signal of bird ID 74799 lost after three years of follow-up, while the monitoring of bird ID 74794 was terminated by the project after 5.5 years of tracking (Tables 1 and 2). Their primary habitat area was in the north-central part of Thailand, and both birds lived around the sizeable fresh marsh (Bungborapet) where rice farms were abundant (Fig. 2a and b).

**Fig. 3:**
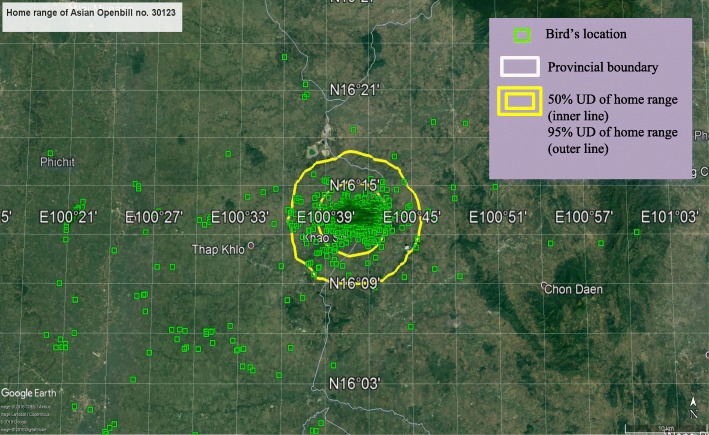
The home ranges of Asian Openbill ID 30123 using satellite telemetry tracking

The Openbill ID number 30123 from Phitsanulok province also foraged around its area of origin. The flock of this Openbill shared the same habitat with great herons in the rice paddies in Phitsanulok province. It moved within an average home range of 360 km/month (range 4.6 to 2833: 95% UD) (Tables 1 and 2). The project terminated the monitoring of this bird after 4.3 years of follow-up. Mostly, the bird moved near the site of satellite tagging (Fig. 3).

### Ecology of foraging areas and foods of Asian Openbills

**Fig. 4:**
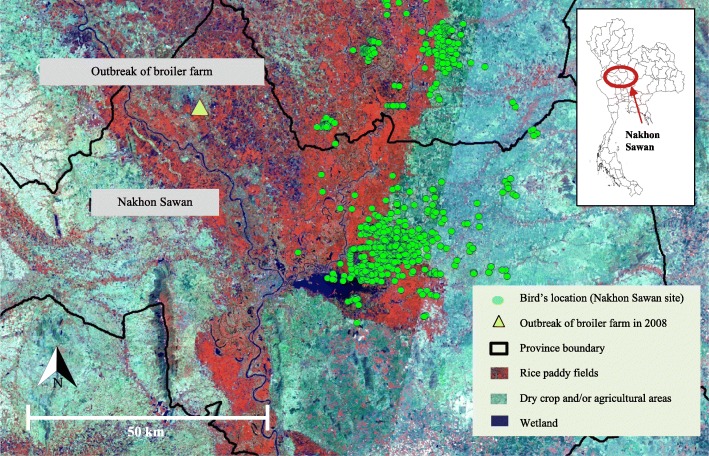
Rice paddy fields, the common habitat of the Asian Openbills as displayed by satellite image (Landsat-7)

The spatial analysis demonstrated that the Openbills foraged in wetlands nearby water reservoirs, rivers, and agricultural areas, in particular rice paddies, in most of the time, and less frequently in dry agricultural lands (Fig. 4). Field observations revealed that the flocks of the Openbills split into small groups during non-breeding season for feeding and resting. Birds formed colonies again for breeding at their original nesting sites in the next November.

The Openbill diet included mollusks, crabs and some insects [5], but their favorite prey was the apple snails such as *P. canaliculata* and *P. insularum.* Field observations also showed that the movements of Openbills were well correlated with the rice crop rotation. Birds moved from one place to the others where new rice crops appeared, and there were plenty of rice sprouts. The apple snails came on for feeding on these young rice stems of age around 10 days, on the other hand, the snails were the favorite food of the Openbills. The land became dry during the harvesting season. In this period, the snails embedded themselves in the mud and lived silently underground. When water returned either through rainfall or irrigation canals, the land became wet and muddy, and it was time to start the new crop of rice. The snails came out of the ground to feed, and snail population attracted the Openbills again.

### Surveillance for H5N1 HPAI infection in Asian Openbills

Blood, together with respiratory and cloacal swab samples, were collected from Openbills between January 2007 and 2010 to monitor the H5N1 HPAI virus infection in flocks of the tracked birds. Testing for the viral genome and isolation of H5N1 HPAI virus from 566 swab samples yielded negative results. However, MN assay of 431 healthy adult storks detected H5N1 antibody titers ≥40 in 2.2% (4 of 181) of serum samples collected in 2007, 1.7% (2 of 120) in 2008, and none in 2009 and 2010 (Table 3).

**Table 3:** Serosurveillance of HPAI H5N1of Asian Openbills in Thailand during 2007–2010

Month	Percentage of HPAI H5N1 antibody-positive samples			
	2007	2008	2009	2010
Jan	–	–	–	0/66 (0%)
Feb	0/48 (0%)	0/57 (0%)	–	–
Mar	0/59 (0%)	–	–	–
Apr	–	–	0/64 (0%)	–
May	–	–	–	–
Jun	–	–	–	–
Jul	–	–	–	–
Aug	–	–	–	–
Sep	0/3	–	–	–
Oct	–	–	–	–
Nov	0/1	–	–	–
Dec	4/70 (5.7%)	2/63 (3.2%)	–	–
Total antibody-positive samples per year	4/181(2.2%)	2/120 (1.7%)	0/64 (0%)	0/66 (0%)

### Movement of Openbills in relationship to AI infection in other wild birds

Role of Openbills in the spread of H5N1 HPAI virus to poultry and other wild birds was explored. The wild birds that lived along the flyways of the tracked flocks were trapped by mist nets. Their blood together with respiratory and cloacal swab samples was collected and investigated for H5N1 HPAI virus infection. MN assay was performed in 206 serum samples collected from 17 bird families. The result showed the MN antibody titers of ≥40 in only four birds: three Streak-eared bulbuls (family Pycnonotidae) from Nakhon Sawan province, and one Ashy wood swallow (family Artamidae) from Nakhon Pathom province in 2008 (Additional file 1). All seropositive birds were healthy adults. The viral genome and the H5N1 HPAI virus could not be detected in 851 birds that were tested.

### Movement of Openbills and AI outbreaks

Positions of the tagged Openbills and their flyways were determined from the signals emitted from satellite transmitters. We sent field epidemiologists to the areas where Openbills foraged to interview the villagers for evidence of AI outbreaks. The interviews revealed no evidence of H5N1 HPAI outbreaks along the flyways of the Openbills during the monitoring period from 2007 to 2013. One AI outbreak occurred in a broiler farm for local consumption in Nakhon Sawan province in November of 2008, but that place did not overlap the habitats of our Openbills (Fig. 4).

## Discussion

**Fig. 5:**
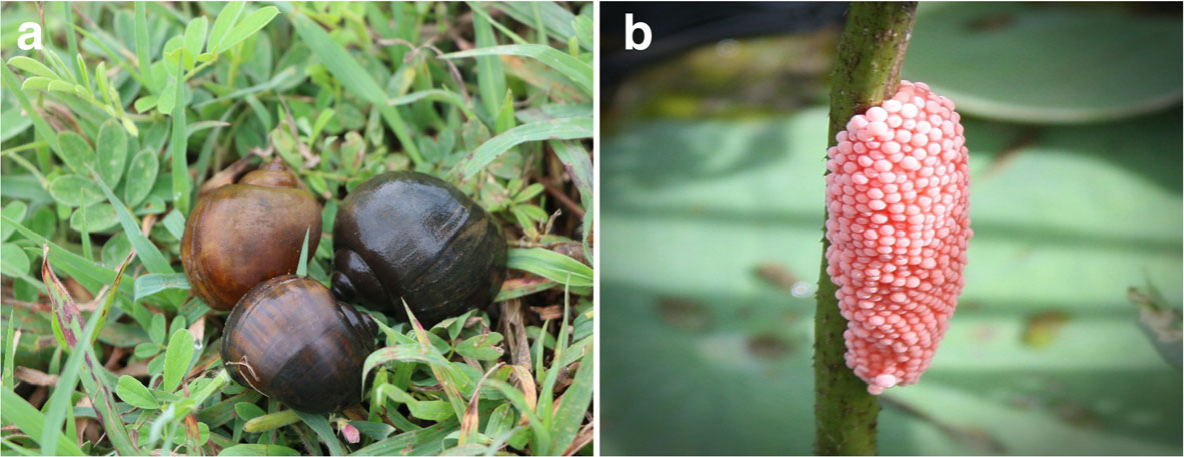
Apple snails (**a**); and a clutch of snail’s eggs (**b**)

The present study determined the potential role of Openbills in the spread of H5N1 HPAI virus by using satellite telemetry to monitor the flyways of birds which overlapped to the locations of AI outbreaks. Using solar power PTT-100 and Argos/GPS PTT-100 transmitters, we monitored the movements of 5 Openbills for periods varying from 1 to more than 5 years between February 2007 and September 2013. During the monitoring period, the flyways or movements of these Openbills did not correlate with the locations of H5N1 HPAI outbreak. We noted that with the strict control measures of the government and private sector actors, the occurrence of AI outbreaks was rapidly declining and became rare events during our study period. Previous investigators have demonstrated an H5N1 virus infection rate of 26.7% in Openbills in 2004; subsequently, the prevalence decreased to 0.9% in 2005, and 0% in 2006 and 2007 [11]. Similarly, the H5N1 HPAI infection rate in the flocks of Openbills in this study was low as demonstrated by the prevalence of H5N1 neutralizing antibody of 2.2% in 2007, 1.7% in 2008, and 0% in 2009 and 2010. Nevertheless, the presence of H5N1 antibody in some Openbills suggested that they might have been infected and survived. Our previous work showed that Openbills in captivity were susceptible to H5N1 HPAI virus infection. All of the virus-inoculated Openbills developed clinical symptoms and died, even with a low virus inoculum of 10 TCID50 [13]. Pigeons, however, were more tolerate [14]. Another group of investigators also reported that the inoculated pigeons did not develop clinical symptoms, even with high virus inoculum of 10^6^ egg infectious dose 50 [15]. Also, the prevalence of the H5N1 HPAI infection rate in a variety of wild birds was approximately 0.27% (17/6263) in the previous Thailand nationwide study from 2004 to 2007 [11], compared with 1.9% (4/206) from 2008 to 2009 as determined by MN assay for H5N1 antibody in this study (Additional file 1). The evidence supported that the most of Openbills would die with AI infection, the survived birds may develop the immunity and may not be a carrier of AI. Therefore, the healthy Openbills may be less likely to shed the virus to the wild birds living in the vicinity of the flyway [11, 13]. We have shown that the movement areas of Openbills were strictly related to populations of apple snails, which coupled with the rice crop rotation [5, 16]. An Asian Openbill can eat as many as 123 apple snails per day. The highest density of snails can reach 2.49 individuals/square meter [5]. This apple snail species (*P. canaliculata* Lamarck) is an alien imported from Taiwan and Japan into Thailand in 1982, for decorating and cleaning the walls of fish tanks. Snails escaped and bred very quickly, becoming overpopulated. They subsequently spread in natural waterways throughout the country. After the big flood in 1995, apple snails were found in at least 60 of 76 (number at that time) provinces of Thailand [7, 16]. In nature, the apple snails live in freshwater marshes, ditches and rice paddies (Fig. 5a). A snail can lay approximately 400–3000 eggs per clutch, and it can lay eggs throughout the wet season (Fig. 5b). During the dry season, apple snails embed themselves underground where they can survive without eating for years. These alien snails now replace most populations of native *Pila* snail species in the fields. Apple snails are an enemy that causes considerable economic losses in rice agriculture. The Openbills partially control them as predators. Eventually, the free-grazing ducks also feed on snails besides falling rice grains after harvesting season.

The primary factor that contributes to the over-population of the apple snails in Thailand is the improvement of the irrigation system and rice farming. Several decades ago, Thai farmers grew rice only once or twice a year depending on rainfall and the water supply. Improvements of the irrigation system by construction of dams, large water reservoirs, and canals, together with rice strain selection, allowed Thai farmers to grow rice all year round. Crop rotation takes place two to three times a year and generates abundant young rice stems, a large food surplus for apple snails. During our monitoring period, the movements of Openbills from their nesting places to feeding areas coincided with the beginning of a new rice crop (Additional file 1). With abundant young rice stems available all year round, the population of apple snails enlarged greatly. Consequently, with plenty of the apple snails available, the Openbills do not need to migrate back to South Asia anymore. Moreover, the Openbill is a protected animal species by Thai law, and this has facilitated their expansion in Thailand in recent years. Initially, there was only a single colony of the Openbills in Thailand, situated at Wat Pailom, Pathum Thani province. At present, there are several colonies throughout the country [17], and they have become permanent residents in Thailand. This result is opposite to our finding on tracking the flyways of brown-headed gulls that they are winter visitors to Thailand until the present [18]. In that study, direct linkage between the flyways of brown-headed gulls and AI spread in Thailand could not be demonstrated. Nevertheless, the gulls flew across 7 countries that faced the problem of H5N1 AI outbreaks. The role of migratory birds on AI spread has been suggested in many studies [19–21].

It is common to see Openbills sharing the same habitats with free-ranging ducks and other resident birds in marshes and rice paddies in Thailand. Improvements in rice farming have caused enormous increases in populations of Apple snails, which has changed the behavior of Openbills from being migrants to becoming permanent residents of Thailand. The Openbills have been found dead in the rice fields along with ducks. We suggest that the ducks might be transmitting the virus to Openbills, as free-ranging ducks have been shown to associate with H5N1 HPAI spread in Thailand [22].

H5N1 HPAI virus was first identified in Guangdong, China, in 1996. The emerged virus has gone through genetic evolution rapidly and resulted in at least 32 clades/subclades at present [23]. Subsequently, multiple events of gene reassortment between H5N1 virus and the other AI virus subtypes haves given rise to the H5 viruses with various N subtypes over time, i.e., H5N2, H5N5, H5N6, H5N8 and H5N9 [24]. The H5N1 viruses that spread all over Thailand since the initial epidemic waves belonged to clade 1 [25], and the virus in clade 2.3.4 was first detected in the northeastern region of Thailand between 2006 and 2007 [26]. The only AI reassortants recognized in Thailand were generated from reassortment of the internal gene segments among H5N1 virus population [27]. Even though our laboratory investigation system can pick up all subtypes of HPAI and LPAI viruses, neither H5Nx nor other AI virus had been detected in this study. Nevertheless, a recent report from the other group of Thai investigators showed that they could isolate an H5N2 LPAI virus from a cloacal specimen collected from apparently healthy free-ranging ducks in 2007 [28]. The H7N9 virus has not been detected in Thailand up to the present.

**Fig. 6:**
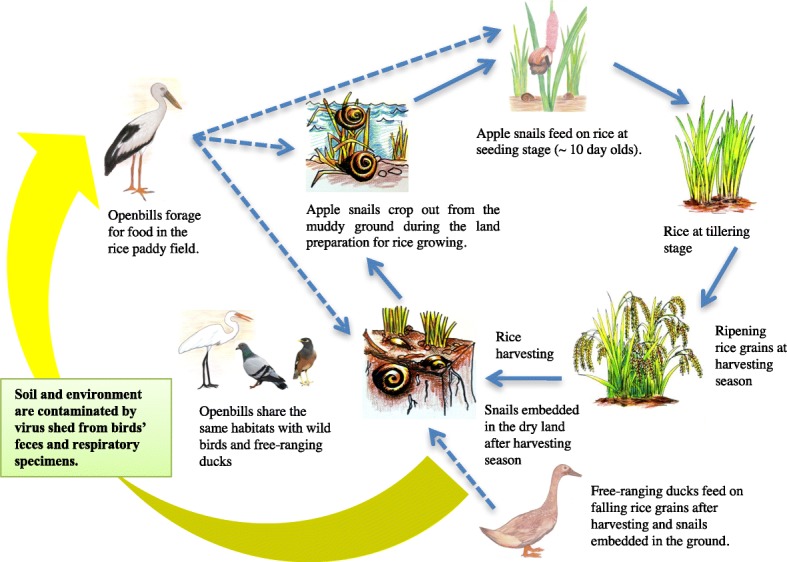
Influence of ecological factors on the life cycle of Asian Openbills and their risk of getting H5N1 HPAI virus infection from free-ranging ducks and other wild birds through habitat sharing

Although the relationship between flyways of Openbills and H5N1 AI spread could not be established in this study, a possible risk of Openbills on acquiring H5N1 infection through sharing habitats with free-ranging ducks has been demonstrated. Many environmental factors affect the behavior of Openbills and their possible role in carrying the H5N1 virus (Fig. 6). During 5 years of our study, the life cycle demonstrating the relationships between the Openbills’ flyways, apple snail populations, free-ranging ducks and wild birds and rice agriculture has been established for the first time (Fig. 6).

## Conclusions

Satellite telemetry had been conducted to monitor the flyways of 5 Asian Openbills in correlation to their potential role on H5N1 AI spread to poultry and domestic birds and within their flocks. The flyway analysis showed that Openbills played no role in AI spread which was probably due to the low prevalence of H5N1 virus during the monitoring period of 6 years. The flyway data showed that the Openbills changed their behavior from being the migratory birds to be the resident birds according to the abundant surplus of the Apple snails, their favorite food and an exotic animal species of Thailand. Moreover, an improvement of irrigation system improved the efficiency of rice farming and made the young rice stem available all year round to feed the Apple snails. Instead of demonstrating the role of Openbills on AI spread, this study first revealed the influence of agricultural and ecological changes on the Openbills life cycle.

## Methods

### Study design

Openbills from three colonies in different geographical areas were tagged with satellite transmitters, and their flyways/movements were monitored until the transmitter signals lost or the project terminated the monitoring. Throughout the monitoring period, the location of each bird tagged was positioned from the transmitter signals emitted. Field investigators trapped the birds in their breeding colonies where the numbers of birds were at highest density. Approximately 50–60 birds per flock were trapped each time for collection of oropharyngeal and cloacal swabs and blood specimens for investigation of H5N1 AI virus infection. The wild birds living in the vicinity where Openbills foraged were also trapped and investigated for H5N1 AI virus infection, in particular, those living in the areas with poultry die-offs, and/or HPAI outbreaks.

### Study sites

Three sites where Openbills nested and formed colonies were located in three provinces of Thailand: Bang Len district, Nakhon Pathom province, in Central Thailand; Bungborapet (a sizeable fresh marsh), Nakhon Sawan province, in the northern Central region; and Phitsanulok province in North Thailand; at distances of 58, 244 and 444 km from Bangkok, respectively. All study sites had good irrigation systems and are close to rivers or water reservoirs. The sites were mainly agricultural areas with paddy rice fields. Two to three rice crops are produced per year. Repeated outbreaks of H5N1 HPAI were reported in several poultry farms in these three provinces during 2004 to 2005.

The colony in Nakhon Pathom province was located in a mango orchard where the Openbills nested on the mango trees. This study site was surrounded by several poultry farms and various kinds of farm gardens including rice fields. The Nakhon Sawan site was close to Bungborapet where hundreds of species of resident and migratory birds shared the same habitat. The third study site, in Phitsanulok province, was surrounded by agricultural fields. This province is famous for the breeding of fighting cocks.

### Tracking the flyways of Openbills using satellite telemetry

**Fig. 7:**
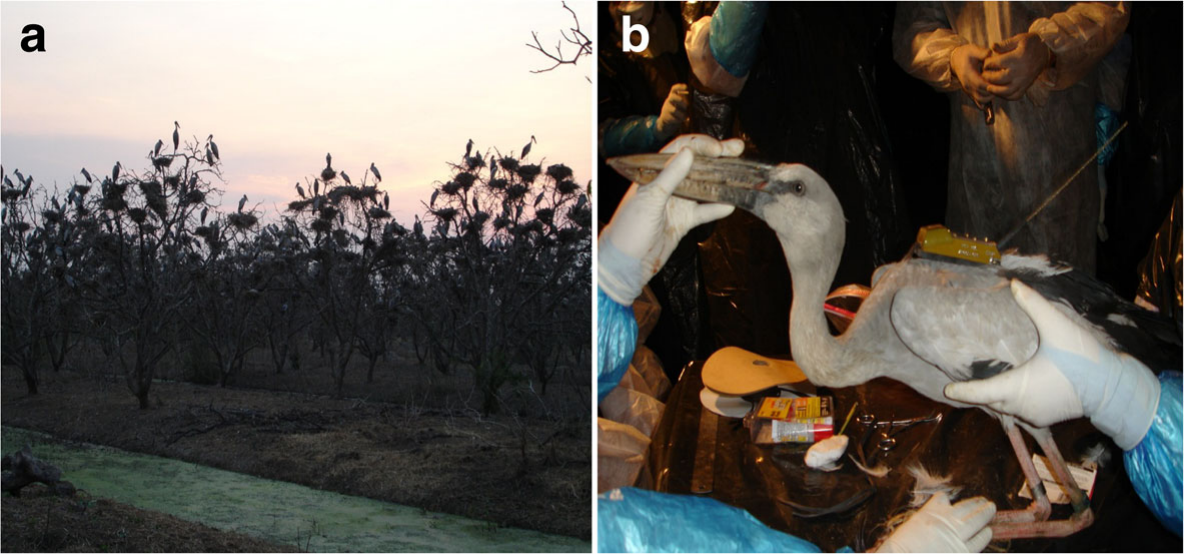
Tagging of the Asian Openbills: (**a**) An overcrowded colony of Openbills with several nests on each tree; (**b**) Tagging an Openbill with a satellite transmitter and a ring band

Asian Openbills were trapped from their nests by hand net during the night. Being colonial breeders, the birds built several nests in the same tree (Fig. 7a). The birds were given physical examinations for health status. Oropharyngeal and cloacal swabs were collected and kept in separate tubes of viral transport media, and 1–2 ml of blood was collected from the wing vein or jugular vein. All birds were screened at the study sites for influenza viral infection by detection of the influenza viral antigen in swab samples using immunochromatography (BioChek, London, UK). Other aliquots of swab samples and blood specimens were sent for further complete investigation of H5N1 HPAI infection at the Virology Laboratory, Faculty of Veterinary Science, Mahidol University. None of the Openbills screened was infected with the H5N1 virus.

Five healthy adult Openbills negative for influenza viral antigen were selected from colonies in the three study sites for flyway monitoring by satellite telemetry. Each of these Openbills was tagged with a ring band and fitted with a 35-g-solar power PTT-100 or a 40-g solar Argos/GPS PTT-100 satellite transmitter (Microwave Telemetry Inc., Columbia, MD) on its back with a Teflon harness (Bally Ribbon Mills, Bally, PA) (Fig. 7b). The solar PTT-100 transmitters operated at a frequency of 401.650 MHz with a standard duty cycle of 10 h on and 24 h off for recharging the batteries. The solar-powered Argos/GPS PTT-100 satellite transmitter was attached to a tiny GPS receiver for locations with higher accuracy [29]. This transmitter package weighed about 0.06% of the birds’ body weights. The birds were freed at the capture sites within 1 h after tagging. The activated satellite transmitters attached to the Openbills emitted ultra-high frequency signals that could be detected by special ARGOS receivers on a polar-orbit weather satellite. The data on locations of the tagged birds were retrieved from the emitted signals, recorded and relayed to a ground station in the United States every two days, and subsequently to the server of Argos CLS Company (Toulouse, France). The data on bird movements at location classes 1, 2 or 3 according to Argos was analyzed in our data manipulation laboratory, and further mapped with Google Earth Program version 7.1.5 (Google, Mountain View, CA, USA) to obtain the real-time location of birds with a precision of less than 1500-m error. If the emitted signals lost for longer than one month, it implied that the transmitter-tagged bird was sick or dead, then the flyway monitoring was terminated.

### Spatial analysis

Microsoft Excel 2016 (Microsoft Corporation, Redmond, Washington, USA) was used for the data analysis. The positions of each Openbill were displayed and analyzed by ArcGIS 9.3.1 and ArcGIS 10 software (Environmental Systems Research Institute, Redlands, CA, USA). More than 10,000 records of the bird positions were cleaned and determined for their habitats and home ranges using a fixed kernel density estimator, and then displayed on Google Earth maps.

### Laboratory investigation

Oropharyngeal and cloacal swabs from Openbills and other wild birds caught during 2007 to 2010 were used for detection of H5N1 HPAI viral genome by real-time reverse transcription-polymerase chain reaction (RT-PCR), and also for virus isolation in chick embryonated eggs and Madin Darby canine kidney (MDCK) cells; and blood specimens were used for detection of H5N1 antibody by microneutralization (MN) assays.

#### Viral genome detection

Real-time RT-PCR for detection of H5N1 HPAI viral genome was performed using the protocol as described by OIE (https://www.oie.int/fileadmin/Home/eng/Health_standards/tahm/2.03.04_AI.pdf) and/or those established by the U.S. Centers for Disease Control and Prevention (CDC). Oropharyngeal and cloacal swabs from each bird were investigated in separate reaction tubes.

#### Viral isolation technique

Cloacal and throat swab specimens were inoculated separately in chick embryonated eggs and Madin Darby canine kidney (MDCK) cell monolayers, in duplicate. Amniotic and allantoic fluids and MDCK culture supernatant were screened for the presence of influenza virus by hemagglutination with 0.5% goose red blood cells. Two blind passages were carried out before reporting the result as negative virus isolation.

#### Microneutralization (MN) assay

ELISA based-MN assay for detection of neutralizing antibody to H5N1 HPAI virus was employed according to the WHO manual for avian influenza [30], and modified by our laboratory [31] in which MDCK cell monolayers were used instead of MDCK cell suspension. The assay was performed in microculture plates in duplicate using A/chicken/Thailand/ICRC-V143/07(H5N1) at a final concentration of 100 tissue culture infective dose 50 (TCID50) as the test virus. Influenza viral nucleoprotein produced in the virus-infected cells was detected by a mouse monoclonal specific antibody (Chemicon International, Inc., Tecumala, CA) as the primary antibody and horseradish peroxidase conjugated-rabbit anti-mouse Ig (Dako Cytomation, Denmark) as the secondary antibody. Antibody titer was defined as the reciprocal of highest serum dilution that causes a 50% reduction in the amount of viral nucleoprotein produced as compared to the virus controls.

## Additional file

Additional file 1: **Table S1.** Serosurveillance in the wild birds along the movement areas of Asian Openbills. **Figure S1.** The rice farming area nearby the large fresh marsh, the major habitat of the Asian Openbills. (PDF 1994 kb)

## Abbreviations

AI: Avian influenza
CDC: The Centers for Disease Control and Prevention
CI: Confidential interval
DLD: The Department of Livestock Development
HPAI: Highly pathogenic avian influenza
km^2^: Square kilometer
MDCK: Madin Darby canine kidney
MN: Microneutralization assays
OIE: The Office International des Epizooties
OLAW: The Office of Laboratory Animal Welfare
RT-PCR: Real-time reverse transcription-polymerase chain reaction
TCID: Tissue culture infective dose
UD: Utility distribution
