# Supplementary material for: Satellite telemetry tracks flyways of Asian Openbill storks in relation to H5N1 avian influenza spread and ecological change
Source: BMC Vet Res. 2018 Nov 16;14:349. doi: 10.1186/s12917-018-1683-x (PMC6240270; doi:10.1186/s12917-018-1683-x)
Supplement: Supplementary file 1 — Table S1. Serosurveillance in the wild birds along the movement areas of Asian Openbills. Figure S1. The rice farming area nearby the large fresh marsh, the major habitat of the Asian Openbills. (PDF 1994 kb) [file 12917_2018_1683_MOESM1_ESM.pdf]

Additional file 1

Table S1. Serosurveillance in the wild birds along the movement areas of Asian Openbills.

**Family**	**Antibody titer**						
	**2008**			**2009**			**Total**
	**≤ 20**	**≥ 40**	**Sum**	**≤ 20**	**≥40**	**Sum**	
Alcedinidae	0	0		1	0	1	1
Anatidae	53	0	53	0	0		53
Artamidae	1	1	2	0	0		2
Ardeidae	13	0	13	2	0	2	15
Columbidae	25	0	25	2	0	2	27
Coraciidae	1	0	1	0	0		1
Cuculidae	4	0	4	2	0	2	6
Dicruridae	2	0	2	1	0	1	3
Jacanidae	1	0	1	0	0		1
Passeridae	2	0	2	8	0	8	10
Phalacrocoracidae	1	0	1	0	0		1
Ploceidae	0	0		20	0	20	20
Pycnonotidae	12	3	15	7	0	7	22
Raillidae	0	0		1	0	1	1
Sturnidae	13	0	13	10	0	10	23
Sylvidae	3	0	3	10	0	10	13
Turdidae	1	0	1	6	0	6	7
Total	132	4	136	70	0	70	206

Streak-eared bulbuls (family Pycnonotidae) from Nakhon Sawan province and 1 Ashy woodswallow from Bang Len (family Artamidae), Nakhon Pathom province were positive for AI antibody in 2008 (Table S1). No seropositive bird has been detected since 2009.

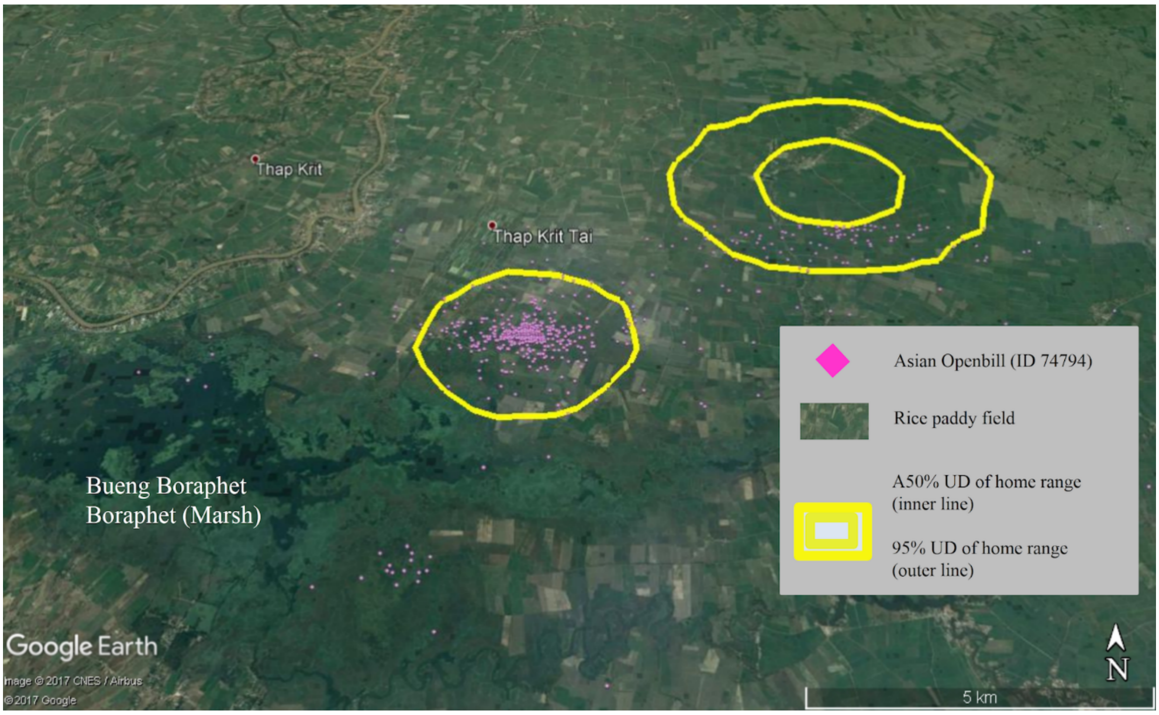

Fig. S1 The rice farming area nearby the large fresh marsh, the major habitat of the Asian Openbills.

The Asian Openbills foraged in the provinces of the top 10 rice farming in Thailand e.g., Phra Nakhon Si Ayutthaya, Suphan Buri, Nakhon Pathom, Chai Nat, Nakhon Sawan, Phichit provinces (data from Department of Agriculture Extension, Ministry of Agriculture and Cooperatives). From the satellite tracking, the birds ID 74794 and 74799 foraged around Nakhon Sawan and Pichit provinces where the rice paddy fields and the apple snails were plenty. The big colonies of Openbills were found around Bueng Boraphet water reservoir in Nakhon Sawan province during the study period (Fig. S1).
